# Microcatheter selective injection technique using ultra low contrast for percutaneous coronary intervention in patients with previous acute kidney injury: a case report

**DOI:** 10.3389/fcvm.2025.1607466

**Published:** 2025-07-10

**Authors:** Arwin Saleh Mangkuanom, Revan Satrio, Brian Mendel, Doni Firman, Nanda Iryuza, Amir Aziz Alkatiri

**Affiliations:** ^1^ Invasive Diagnostic and Non Surgical Intervention Division, Department of Cardiology and Vascular Medicine, Faculty of Medicine, Universitas Indonesia, Jakarta, Indonesia; ^2^ Department of Cardiology and Vascular Medicine, Faculty of Medicine, Universitas Indonesia, Jakarta, Indonesia

**Keywords:** AKI, case report, chronic total occlusion, low-dose contrast, microcatheter injection, normal saline, percutaneous coronary intervention

## Abstract

**Background:**

Contrast-induced nephropathy (CIN) is a risk in angiographic procedures, especially for patients with acute kidney injury (AKI). To mitigate this risk, ultra-low contrast percutaneous coronary intervention (ULC-PCI) has been developed, which minimizes the use of contrast agents.

**Case summary:**

A 54-year-old woman with a history of AKI from a prior percutaneous coronary intervention (PCI) was found to have coronary artery disease with three vessels disease and chronic total occlusion in the right coronary artery. To minimize contrast use, she underwent ultra-low contrast PCI using the “microcatheter injection” technique, with only 5 cc of contrast used during the procedure. At one-year follow-up, the patient's LVEF improved from 33% to 56%, symptoms resolved with no chest pain. Her estimated glomerular filtration rate (eGFR) showed no significant decrease, since serum creatinine increased slightly from 1.59 mg/dl to 1.61 mg/dl. and eGFR decreased from 39 to 38 ml/min/1.73 m^2^ in 72 h.

**Conclusions:**

The microcatheter injection technique may serve as a viable strategy for percutaneous coronary intervention (PCI) in patients with eGFR < 30 ml/min/1.73 m^2^ or history of contrast-induced nephropathy.

## 1 Introduction

Contrast-induced nephropathy (CIN) is a potential complication associated with the use of iodinated contrast agents in angiographic procedures, particularly among patients with acute kidney injury. To mitigate this risk, ultra-low contrast percutaneous coronary intervention (ULC-PCI) has been developed, which minimizes or eliminates the use of contrast agents. Critical procedural steps, including catheter engagement, vessel wiring, and stent deployment, are performed using contrast-free techniques (1, 2). We utilized a microcatheter selective injection technique using ultra low contrast that facilitates ULC-PCI. This case report outlines the microcatheter injection technique that renders ULC-PCI feasible in these patients.

## 2 Case illustration

A 54-year-old female patient with hypertension, diabetes, and menopause as risk factors for coronary artery disease came to the outpatient clinic. Her chief complaint was chest pain during physical activity. The patient had history of acute kidney injury from previous PCI. Physical examination showed blood pressure of 133/71 mmHg, heart rate of 67 bpm, and oxygen saturation of 98%. The patient exhibited no fever, signs of heart failure, or bronchospasm. ECG revealed inverted T waves at V4-V6, I, aVL, and echocardiography showed mild mitral regurgitation, mild tricuspid regurgitation, akinesia at basal-apical of inferior and basal-mid of inferolateral segments; other segments were hypokinetic, with LVEF 33% and tricuspid annular plane systolic excursion of 15 mm. Even though the echocardiogram revealed akinesia in the inferior and infero-lateral walls, another imaging test was not considered to assess viability due to administration problems.

Coronary angiography from previous hospitalization showed three-vessel coronary artery disease involving the left main artery, along with a chronic total occlusion (CTO) of the right coronary artery (RCA). Laboratory examination showed a decrease in renal function with serum creatinine of 1.59 mg/dl and an eGFR of 39 ml/min/1.73 m^2^. The patient had been discussed in a surgical conference and was scheduled for coronary artery bypass grafting. However, intra-aortic balloon pump support was not available. As the patient continued to experience refractory chest pain, we proceeded with percutaneous coronary intervention (PCI) by implanting two drug-eluting stents (DES) in the left anterior descending artery, which was identified as the initial culprit lesion. Due to presence of chest pain despite anti-anginal treatment with carvedilol 12.5 mg b.i.d and nitrokaf retard 5 mg b.i.d., we also decided for PCI of RCA CTO.

**Figure 1:**
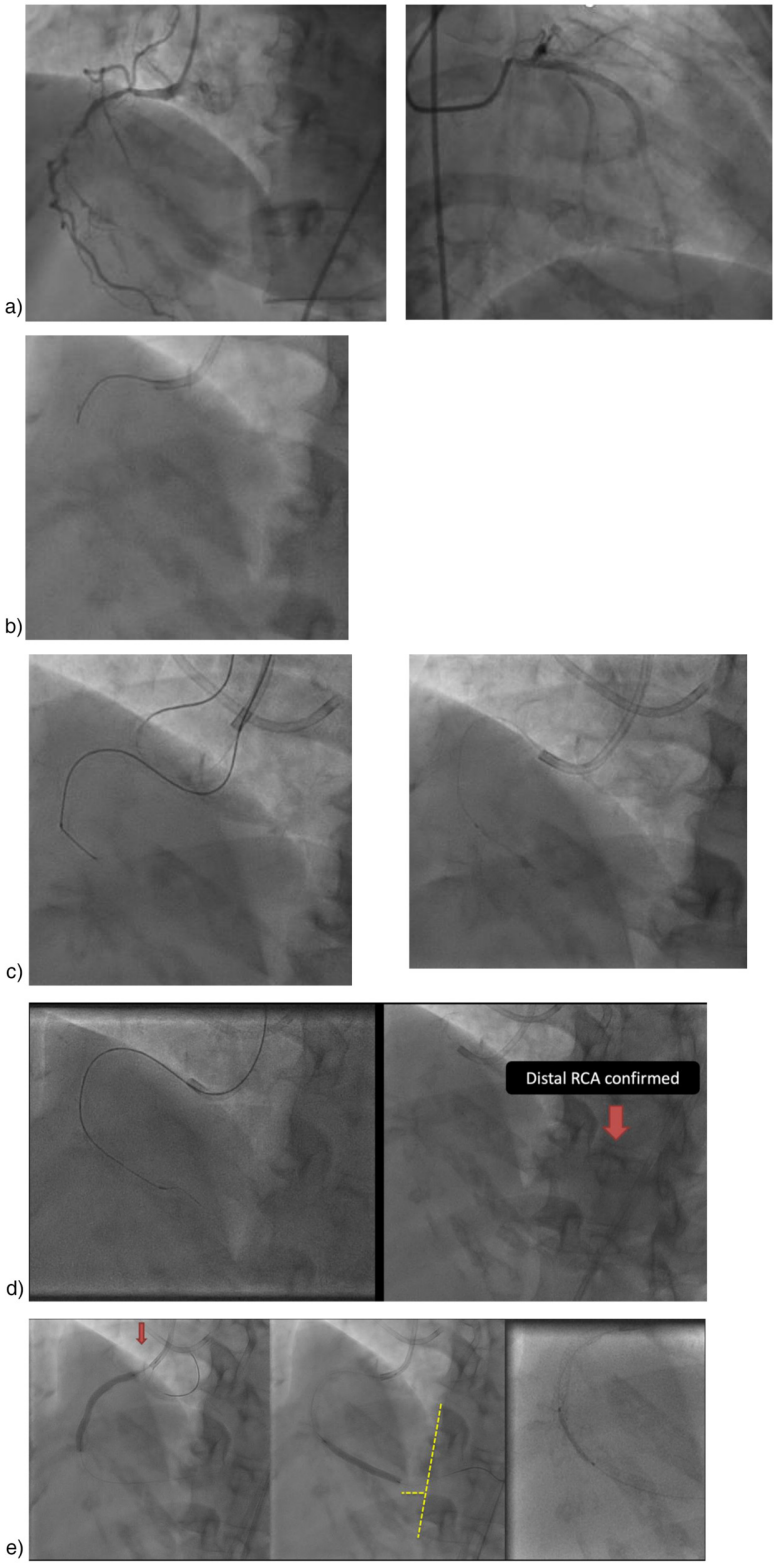
Ultra-Low contrast PCI with microcatheter injection. **(a)** Initial angiography (3 months earlier), **(b)** Cannulation and then using saline 10–20 cc and 0.5 cc contrast, **(c)** A Judkins left catheter can be seen in addition to the right catheter, used to confirm the position of the contralateral wire, if needed. Injections were only made through the right microcatheter. Wiring using Fielder XTA, and confirmed with 0.5 cc contrast for confirmation stump, **(d)** Micro tip injection, **(e)** Stent placement.

We opted for treatment using ultra-low contrast PCI with “microcatheter injection” technique to address the presence of chronic total occlusion (CTO) and minimize contrast use (see Figure 1a). Right coronary artery (RCA) cannulation was performed using a Judkins Right 3.5/7F catheter, assisted by Finecross 1.8F microcatheter. The microcatheter was placed prior to the lesion before contrast injection. We injected 10–20 ml of 0.9% saline through the catheter and the amplitude of the T-wave inversion increased. Then, runthrough NS floppy coronary wire was inserted. A hand injection of 0.5 cc of contrast confirmed total occlusion in the proximal segment (see Figure 1b). The coronary wire was anchored to the conus branch. Subsequently, an escalation wire (Fielder XT), was utilized to penetrate the lesion and was positioned distally with the backup microcatheter. After removing the wire, another hand injection of 0.5 cc of contrast was performed to verify that the microcatheter was within the RCA lumen (see Figures 1c,d). Predilatation was conducted multiple times with Sapphire balloons measuring 2.0 × 15 mm and 3.0 × 15 mm, advancing from the proximal to the distal segment.

**Figure 2:**
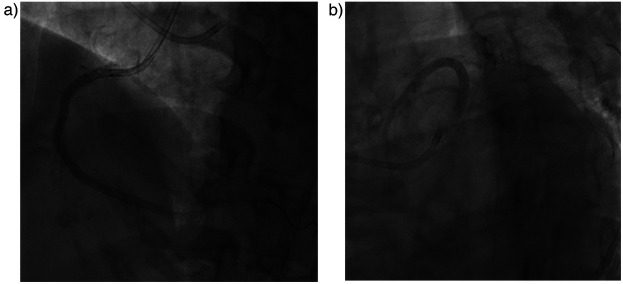
Post PCI angiography evaluation. **(a)** RCA stent and **(b)** LAD stent were in good position.

An anatomical marking technique was employed for the placement of both the distal and proximal landing zones. For the distal landing zone we utilized vertebrae as landmarks, while the proximal landing zone was guided by positioning an additional wire in the conus, allowing precise identification of the ostial location. A drug-eluting stent (DES) Promus Premiere 3.5 × 38 mm was implanted from the ostium to the mid segment, followed by the implantation of a mid to distal DES Xience Xpedition 3.0 × 38 mm. Balloon measurement was performed to ascertain the length of the DES in the mid region by evaluating the gap between the previously placed stents. Subsequently, a third DES Promus Premiere 3.5 × 16 mm was implanted in the mid-right coronary artery (RCA) (see Figure 1E). Angiographic evaluation was conducted using a hand injection of 0.5 cc of contrast (Figure 2), with the total contrast volume utilized during the procedure being only 5 cc. At one-year follow-up, our patient demonstrated a significant improvement in left ventricular ejection fraction (LVEF), increasing from 33% to 56%. Additionally, her symptoms resolved, with no reported chest pain. This patient showed no AKI. There was no significant decline in renal function, as the serum creatinine increased only slightly from 1.59 to 1.61 mg/dl and the estimated glomerular filtration rate (eGFR) decreased minimally from 39 to 38 ml/min/1.73 m^2^ over 72 h.

## 3 Discussions

Percutaneous coronary intervention (PCI) is a well-established and widely utilized treatment for coronary artery disease (CAD) (3, 4). However, one major concern is the risk of CIN (1). We calculated the Mehran score for this patient, identifying key risk factors such as diabetes, an eGFR of 39 ml/min/1.73 m^2^, and the use of 5 cc contrast. The patient's Mehran score was determined to be 7 points, corresponding to a 14% risk of post-PCI contrast-induced nephropathy (CIN) and a 0.12% risk of requiring dialysis due to post-PCI CIN (5).

The CONSaVE-AKI study (2022) (6) demonstrated a significantly higher incidence of contrast-induced acute kidney injury (CI-AKI) in patients undergoing conventional percutaneous coronary intervention (PCI) compared to those treated with the ultra-low contrast PCI (ULC-PCI) approach [17.1% vs. 0%; *p* = 0.012]. The volume of contrast used was substantially lower in the ULC-PCI group (41.02 ± 9.8 ml vs. 112.54 ± 25.18 ml; *p* < 0.0001). Key findings from the study include: (1) a significantly lower rate of CI-AKI in the ULC-PCI group, (2) successful implementation of the ULC-PCI protocol regardless of lesion complexity, (3) comparable safety and efficacy between ULC-PCI and conventional PCI, with no differences in secondary safety outcomes, and (4) in patients with acute coronary syndrome and pre-existing renal dysfunction, PCI was associated with improvement in glomerular filtration rate (GFR).

Ultra-low contrast percutaneous coronary intervention (ULC-PCI), defined as a contrast volume-to-estimated glomerular filtration rate (eGFR) ratio of less than 1, was initially proposed for patients with chronic kidney disease (CKD) who are at elevated risk of contrast-induced nephropathy (CIN) (1). The first pivotal study evaluating the feasibility of this approach was conducted by Ali et al. (7), involving 31 patients with advanced CKD. Subsequently, Rozenbaum et al. (8) performed ULC-angiography in 30 patients, followed by ULC-PCI, without intravascular ultrasound (IVUS), in 16 of them. None of the patients developed contrast-induced acute kidney injury (CI-AKI), marking these studies as the first to demonstrate both the feasibility and cardiovascular safety of ULC-PCI.

### 3.1 Catheter engagement without contrast administration

Studies have demonstrated that intravascular ultrasound (IVUS) not only reduces contrast volume but also lowers the risk of contrast-induced nephropathy (CIN), making it particularly valuable for patients with renal impairment. IVUS-guided percutaneous coronary intervention enables more accurate lesion assessment and optimal stent deployment, which has been consistently associated with lower rates of target lesion failure and stent-related complications. By highlighting these dual benefits, enhanced procedural safety and improved long-term outcomes, IVUS emerges as an essential tool in modern interventional cardiology practice (9, 10).

In ULC angiography, the use of smaller catheters without side holes (5–6 Fr) is recommended to maximize vessel opacification (2). Techniques to minimize contrast include utilizing coronary calcium to confirm catheter engagement. In a study conducted by Kim et al., 0.9% saline or 5% dextrose was injected into the left coronary artery prior to assessing heart rate, QT interval, and T-wave amplitude. The administration of 10–20 ml of 0.9% saline through the catheter allowed for confirmation of appropriate catheter positioning, as evidenced by T-wave inversion or augmentation, along with accompanying ST-segment depression or elevation (9). For diagnostic angiography, using minimal projections and specific angles is essential for optimal visualization. While diluted contrast allows for more imaging runs, it may reduce luminal opacification. Increasing frame rates can help enhance coronary anatomy visualization (3, 4).

**Figure 3:**
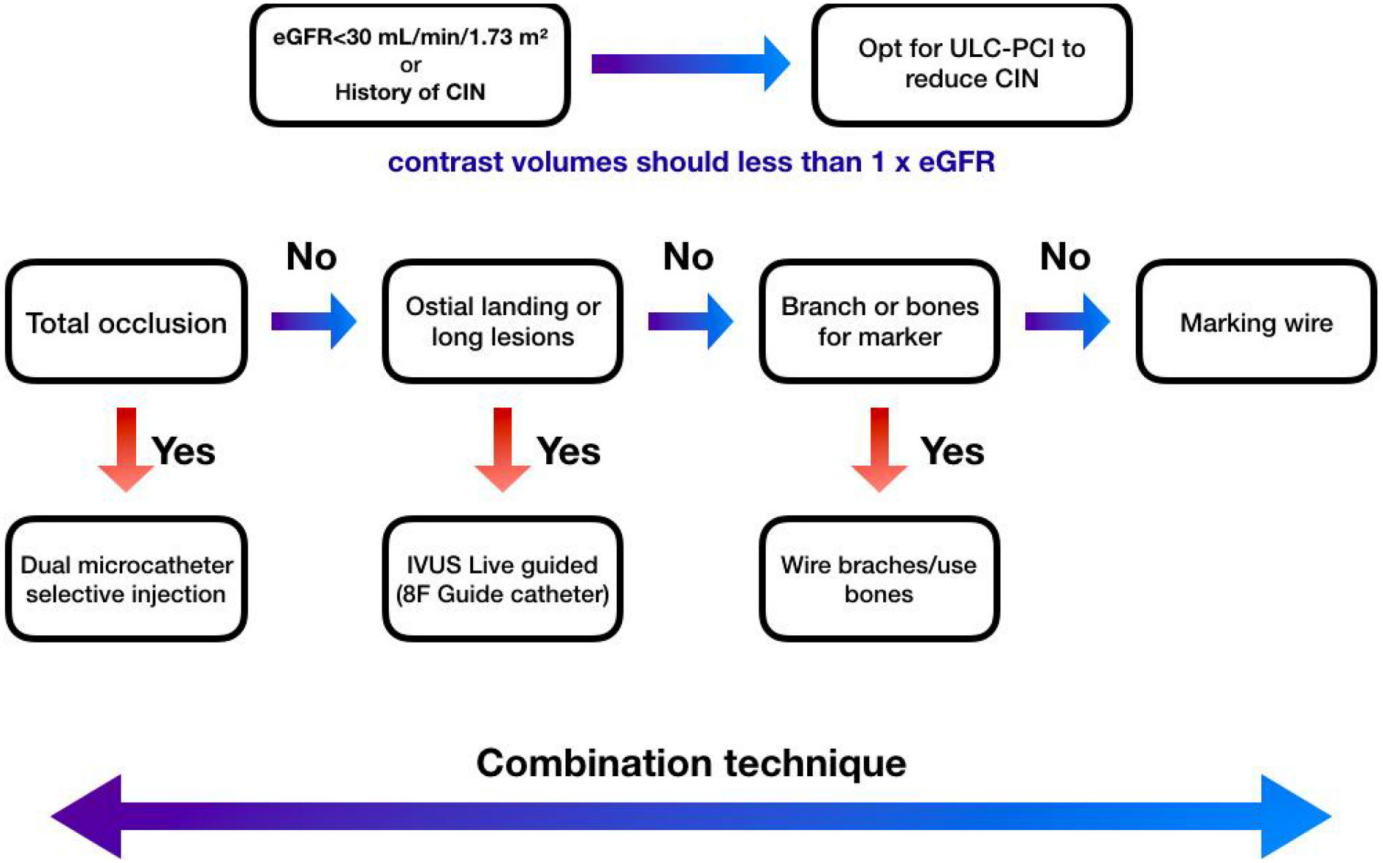
Mangkuanom's algorithm of ultra low-dose contrast percutaneous coronary intervention technique. In patients with an eGFR <30 ml/min/1.73 m^2^ or history of CIN, we opt for an ultra-low contrast percutaneous coronary intervention (PCI) strategy, adhering to the widely accepted definition of ultra-low contrast PCI as a contrast volume-to-eGFR ratio of less than 1. This approach is specifically indicated for individuals with advanced chronic kidney disease who are at heightened risk of developing post-procedural contrast-induced nephropathy (CIN). Notes: eGFR, estimated Glomerular Filtration Rate; IVUS, intravascular ultrasound; ULC-PCI, ultra low-dose contrast percutaneous coronary intervention.

We performed ULC-PCI using a microcatheter, balloon catheter measurement to determine the appropriate length for drug-eluting stent (DES) implantation, and final angiographic assessment with hand-injected contrast. An algorithm was developed to guide technique selection in ULC-PCI(see Figure 3). Patients with an eGFR below 30 ml/min/1.73 m^2^ or a history of contrast-induced nephropathy (CIN) or acute kidney injury (AKI) were prioritized for this approach. Total occlusion dictated the use of microcatheter injection, while its absence led to further assessment of lesion location. If the lesion was ostial or required precise stenting, live-guided IVUS was preferred. Anatomical landmarks or wire branching were used for landing zone identification. Marking the wire served as the fundamental technique, though various methods were combined as needed during the procedure.

## 4 Conclusions

The microcatheter injection technique may represent a feasible and effective strategy for percutaneous coronary intervention (PCI) in patients with acute kidney injury. By enabling revascularization with minimal use of contrast medium, this approach can achieve favorable outcomes while reducing the risk of procedure-related complications.

## Data Availability

The datasets presented in this article are not readily available because of ethical and privacy restrictions. Requests to access the datasets should be directed to the corresponding author.
